# Current research toward optimizing dosing of first-line antituberculosis treatment

**DOI:** 10.1080/14787210.2019.1555031

**Published:** 2018-12-12

**Authors:** Helen McIlleron, Maxwell T Chirehwa

**Affiliations:** Division of Clinical Pharmacology, Department of Medicine, University of Cape Town, Cape Town, South Africa

**Keywords:** Ethambutol, pharmacogenetic, pharmacokinetic, pyrazinamide, rifampin

## Abstract

**Introduction**: Drug concentrations in tuberculosis patients on standard regimens vary widely with clinically important consequences.

**Areas covered**: We review the available literature identifying factors correlated with pharmacokinetic variability of antituberculosis drugs. Based on population pharmacokinetic models and the weight, height, and sex distributions in a large data base of African tuberculosis patients, we propose simplified weight-based doses of the available fixed dose combination(FDC) for adults with drug susceptible tuberculosis. Emerging studies will support optimized weight-based dosing for children. Other sources of important pharmacokinetic variability include genetic variants, drug-drug interactions, formulation quality, and methods of preparation and administration.

**Expert commentary**: Optimized weight band-based dosing will result in more equitable distribution of drug exposures by weight. The use of high doses of isoniazid in patients with drug-resistant tuberculosis would be safer and more effective if a feasible test was developed to allow stratified dosing according to acetylator type. There is an urgent need for more suitable formulations of many second-line drugs for children. The adoption of new technologies and efficient FDC design may allow further advances for patients and treatment programs. Lastly, current efforts to ensure adequate quality of antituberculosis drug products are not preventing the use of substandard products to treat patients with tuberculosis.

## 1. Introduction

Many antituberculosis drugs exhibit wide pharmacokinetic variability in patient cohorts [1–13]. Studies in patients with drug-susceptible tuberculosis (DS-TB) on a standard regimen of rifampin and isoniazid for 6 months with pyrazinamide and ethambutol for the first 2 months, suggest that such variability is clinically important. Specifically, patients with relatively low systemic exposures to rifampin and pyrazinamide have worse treatment outcomes [4,6], while rifampin, isoniazid and pyrazinamide exposures are related to the decline in bacterial load in the sputum during the weeks following initiation of treatment [6,8,14–18]. For ethambutol and isoniazid, as well as second-line drugs for drug-resistant tuberculosis (DR-TB), such as linezolid, cycloserine, moxifloxacin, aminoglycosides, and capreomycin toxicity, are dose-related. Dose optimization should therefore aim to limit the number of patients with lower or higher than optimal drug exposures.

For rifampin and rifapentine, plasma exposures higher than those achieved with current doses result in improved antituberculosis activity and appear to be relatively safe. Recent and ongoing clinical studies are likely to broadly redefine the optimal dosing strategies for these rifamycins and other drugs in treatment and preventive therapy regimens [19–24]. Evidence is needed to inform appropriate doses for tuberculosis at sites where penetration of the drugs into the diseased compartments is limited such as tuberculous pericarditis and tuberculous meningitis [25–27]. For some antituberculosis drugs, dose adjustments are indicated in patients with renal or hepatic impairment. Therapeutic drug concentration monitoring may have a role in some settings and is reviewed elsewhere as part of the approach to personalized therapy for DR-TB [28,29]. These topics are not included in this review.

We focus on the first-line antituberculosis drugs while recognizing the even greater need for evidence to support optimized drug doses and combinations in the emerging regimens for DR-TB, for which current treatment is less effective and more toxic than the first-line regimens. We discuss pharmacokinetic variability in clinical cohorts due to the manufacturing process and storage conditions, patient factors (weight and body composition, other clinical characteristics, and pharmacogenetics) drug-drug interactions, and dose preparation and administration (Table 1). We identify sources of pharmacokinetic variability in adults and children, which could be used to limit systemic pharmacokinetic variability. We suggest revised a priori dose adjustments based on weight to correct the systematic under-dosing of low weight patients with DS-TB.

**Table 1.** Summary of sources of variability discussed in this article, with potential actions that could reduce pharmacokinetic variability.

Source of pharmacokinetic variability (section)	Potential actions mitigating pharmacokinetic variability
Drug formulation (2)	Independent bioequivalence testing against established standard comparator product. Quality surveillance of drug products used in programs. Development and implementation of protocols for regular testing of relative bioavailability in programs. Development of improved *in vitro* screening methods that predict bioavailability, for use in programs.
Weight and body composition (3)	Optimize pragmatic weight band-based dosing guidelines based on contemporary knowledge and pharmacokinetic evidence in adult and pediatric patient populations.
Other clinical covariates (4)	Pharmacokinetic studies to optimize dosing in infants, pregnant women, patients with renal or hepatic impairment, and other special populations.
Pharmacogenetic variation (5)	For clinically important genetic variants, development of field-friendly genetic tests to facilitate dosing by genotype. Population surveys to optimize dosing for the population based on the prevalence of clinically important genetic variants.
Drug-drug interactions (6)	Drug-drug interactions need to be considered and studied within antituberculosis regimens and between antituberculosis drugs and other commonly administered drugs. Studies should preferably be performed in patients. For clinically important pharmacokinetic drug-drug interactions, dose adjustment strategies should be evaluated in patients.
Dose preparation and administration (7)	Investment in the development of user- friendly formulations for children and adults with robust bioavailability under field conditions. The clinical importance, in the context of patients under field conditions, of food effects and other factors influencing bioavailability should be evaluated and if important should be addressed e.g. by developing formulations less vulnerable to food effects.
Laboratory error (8)	Laboratory participation in proficiency testing. Development of platforms for sharing assay methodologies and expertise.

## 2. Drug formulation

A recent metanalysis found that rifampin’s area under the concentration-time curve (AUC) varied 3- to 4-fold between different studies [30]. While between-laboratory differences, and genetic, environmental and pathophysiological differences between the study populations may play a role, evidence from multiple studies suggests that differences in bioavailability between formulations and batches could be responsible for large bioavailability deviations.

An early study of 4-drug FDCs distributed on the global market for the first-line treatment of tuberculosis found that seven of the ten products were not bioequivalent for rifampin [31]. Similarly, a more recent study from China found that four of five FDCs failed bioequivalence criteria for rifampin [32]. Observational studies in patients have attributed massively compromised bioavailability to product or batch quality [12,14,33–36]. Recently, a 20% reduction in the bioavailability of rifampin was described in the 4-drug FDC used in approximately 450 000 South African patients annually [37]. The implications of such smaller changes in formulation quality on treatment outcomes and the development of resistance are unknown, however given the emerging evidence of the importance of exposure-response relationships described for rifampin and other drugs, these compromises may be devastating when they occur on such a large scale [4,6,8].

Although the formulation of rifampin is challenging particularly in FDCs, quality issues appear to occur commonly for other tuberculosis drugs [38]. While quality may be related to a specific product, batch-to-batch variability occurs and storage conditions are also important [12,14,39]. Hence it is clear that in spite of GMP inspections and prequalification programs, products and product batches of inferior quality are on the market and contribute substantially to variability in drug exposure.

## 3. Weight and body composition

Dosing guidelines for antituberculosis drugs typically advocate a uniform milligram per kilogram of body weight (mg/kg) dose, and the drugs are typically dosed by weight band to achieve a narrow mg/kg range using the available formulations [40,41]. A consistent finding of pharmacokinetic studies in adults and children dosed in this way is that lower weight patients have lower drug exposures [5,10,13,18,33,37,42–44]. This can be attributed to the nonlinear relationship between clearance and size whereby smaller people need higher maintenance doses per kilogram of body weight [45]. Weight band doses should be optimized accordingly. Moreover, fat-free mass is frequently more closely related to clearance and volume of distribution than total body weight [7,9–14]. For these drugs (including rifampin, isoniazid, and pyrazinamide), wasted or stunted individuals have lower drug exposures due to a higher proportion of their total body weight being accounted for by fat-free mass. Several clinical studies report low body mass index or low weight as a risk factor for poor tuberculosis treatment outcomes [46–48]. While this association is likely to be multi-factorial, the potential contribution of low drug exposures in wasted and low-weight individuals should not be ignored.

For some drugs like moxifloxacin and clofazimine dose adjustment is limited by the available formulations. The same dose is therefore administered to patients across a wide weight range. Because they receive lower mg/kg doses with this approach, exposures tend to be lower in heavier patients, in contrast to the exposures when a uniform mg/kg dosing approach is used [49]. For clofazimine which takes months to reach steady-state pharmacokinetics and is highly lipophilic, plasma exposures are inversely correlated with percentage body fat [50]. This is in keeping with extensive distribution to the tissues. Heavy patients who are also obese are therefore likely to have relatively low blood concentrations of clofazimine.

### 3.1. Adults

We recently evaluated the population pharmacokinetics of rifampin, isoniazid, pyrazinamide, and ethambutol by weight band in tuberculosis patients treated according to the widely implemented World Health Organization(WHO) recommendations using fixed dose combination(FDC) tablets [5,10,13,37,32]. The pharmacokinetic models for rifampin and pyrazinamide were published previously [10,13]. The models for isoniazid and ethambutol are presented as supplementary material. Fat-free mass was more closely associated than total body weight with clearance and volume of distribution for rifampin, isoniazid and pyrazinamide. For each drug, markedly lower drug exposures are evident in the lower weight bands (Figure 1, left panel). We used simulations to predict the number of 4-drug FDC tablets (using the widely available rifampin/isoniazid/pyrazinamide/ethambutol 150/75/400/275 mg FDC) to achieve the most equitable drug exposures across weight bands. The simulations were based on the weight, height and sex distributions among 1092 African patients with DS-TB in our database. Given the large proportion of patients with drug concentrations low by comparison to the recommended ranges [51], together with evidence for poor treatment outcomes with low drug exposures and concern that low drug exposures might drive resistance, as well as the vast experience of the safety of doses in the 55–70 kg band, the exposures achieved in this 55–70 kg weight band provided the target for the simulations. Between patient variability was reduced and dosing simplified by adding one FDC tablet for patients under 55 kg (Figure 1, right panel). Very similar results were obtained when a similar approach was applied to these drugs based on data from Uganda [33]. The results of these studies confirm that higher mg/kg doses are needed in patients in the lower weight bands, and guidelines should be revised to avoid systematic underdosing of low weight patients.

**Figure 1.**
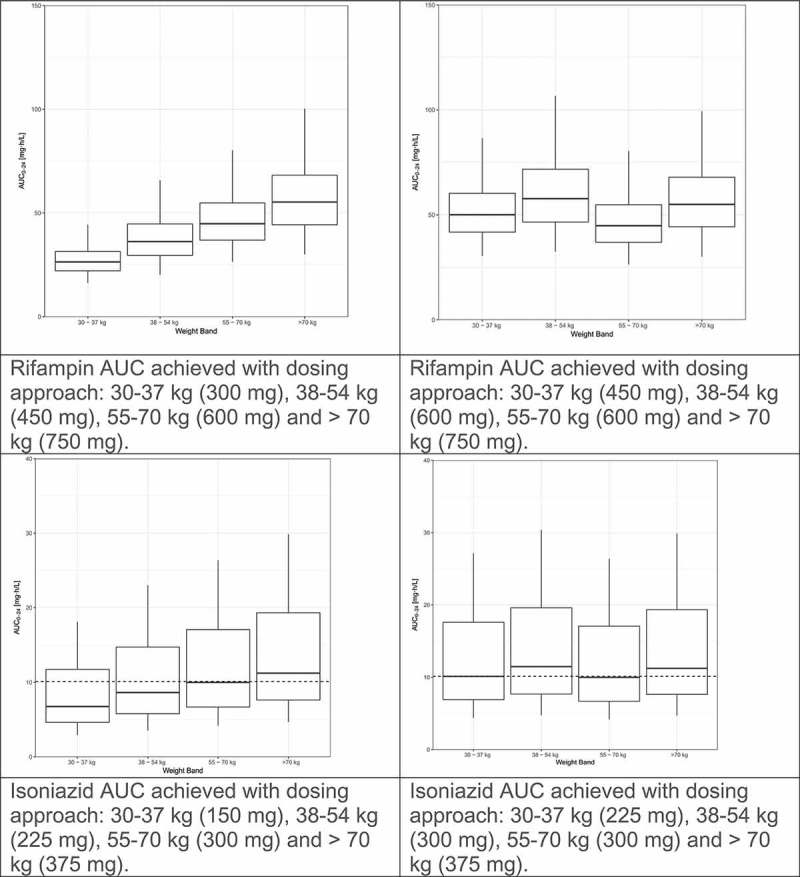
Evaluation of WHO’s current weight band-based doses for the treatment of drug-sensitive TB. AUC to 24 h (AUC_0–24_) for rifampin (Court R, Chirehwa MT, Wiesner L, Wright B, Smythe W, Kramer N, McIlleron H. Quality assurance of rifampin-containing fixed-drug combinations in South Africa: dosing implications. Int J Tuberc Lung Dis. 1 May 2018;22(5):537–543. Reprinted with permission of the International Union Against Tuberculosis and Lung Disease. Copyright © The Union), isoniazid, pyrazinamide and ethambutol, by weight band. Left-hand panel: Predicted AUC_0–24_ when the currently recommended fixed dose combination tablets (rifampin/isoniazid/pyrazinamide/ethambutol 150/75/400/275 mg) are given according to the dosing guidelines for adults (<38 kg—2 tablets; 38–54.9 kg—3 tablets; 55–70 kg—4 tablets; >70 kg—5 tablets). Right-hand panel: Predicted AUC_0–24_ when patients <55 kg are given an additional FDC to account for the higher CL/kg in smaller individuals (<38 kg—3 tablets; 38–54.9 kg—4 tablets; 55–70 kg—4 tablets; >70 kg—5 tablets).

**Figure 1.**
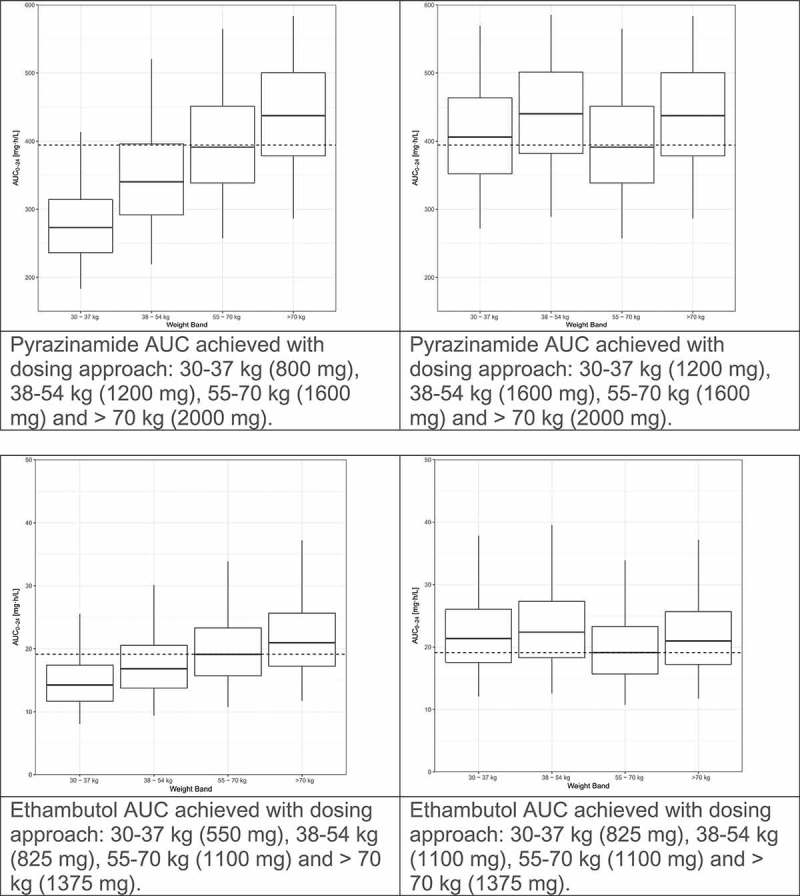
(Continued).

### 3.2. Children

Although many children have minimal disease, young children are particularly vulnerable to severe forms of tuberculosis and childhood mortality is estimated by the WHO to be as high as 25%. Due to growth and development, drug exposures tend to be even more variable and less predictable among young children than they are in adults [18,44,52–55]. Until recently optimization of tuberculosis treatment has been neglected in children. In response to several small studies showing lower drug exposures in children than in adults treated with the same mg/kg doses, the WHO revised dosing guidelines for children under 12 years of age in 2010 [56]. The doses of have been increased by 50% for rifampin to 15 (10–20) mg/kg and by 100% for isoniazid to 10 (7–15) mg/kg, while 35 (30–40) mg/kg doses of pyrazinamide are recommended. Emerging studies evaluating the revised doses show that while the higher pediatric weight bands achieve average rifampin and pyrazinamide exposures similar to adults, in children under 12 kg exposures fall short of those in adults [42–44,55]. Early results from the SHINE study (ISRCTN63579542) show a median rifampin AUC for children in the 4–7.9 kg weight band, half that for children in the 16–24.9 kg band [44]. Therefore, in young children the higher mg/kg doses appear to be insufficient for rifampin and pyrazinamide. Much of the disparity between the higher and lower weight bands is due to the higher clearance per kilogram of body weight in smaller individuals [45]. For rifampin, however, reduced bioavailability has been shown to contribute to low exposures in young children; in spite of reduced clearance due to immaturity of drug disposition pathways, the youngest had lowest rifampin exposures among children treated with a stringent regulatory authority-approved formulation [42]. Children over 25 kg who are treated with the lower adult mg/kg doses also have disturbingly low drug exposures [44,57]. Recent evidence linking low rifampin exposure in children to treatment failure and death emphasizes the urgency to optimize pediatric dosing [18]. Emerging studies support improved weight-based dosing of the first- and many second-line drugs for children [53,58–61], however further pharmacokinetic studies in infants and children are needed to represent different geographical regions and account for age, weight and formulation-related effects.

## 4. Other clinical characteristics

While optimized dosing by weight results in markedly more equitable exposures across the weight bands, much of the pharmacokinetic variability between individuals remains. HIV is a common co-infection in patients presenting with tuberculosis. Several studies have investigated the association of HIV infection with the pharmacokinetics of the first-line drugs for DS-TB [2,21,62–65]. The findings across different studies are not consistent. This may be due to methodological differences between the studies, or differences between the study populations such as disease severity and wasting, inflammatory and immune status, and the presence or absence of concomitant antiretroviral treatment [11,66–68]. Reduced antituberculosis drug exposures have also been described in males compared to females [1,12]. Confounding by increased clearance per kilogram of fat-free mass [7,9–14], may contribute to the association of low drug exposures with HIV infection and male sex in some cohorts. Among Tanzanian tuberculosis patients with HIV infection, nutritional supplementation during the first 2 months of treatment improved the bioavailability of rifampin [7]. The effect was independent of fat-free mass, suggesting a direct effect of the nutritional intervention on the absorption of rifampin. As rifampin is highly protein bound, increased albumin levels with improved nutrition, may have contributed to an apparent increase in bioavailability [69]. Changes in body composition and plasma proteins have been implicated to alter pharmacokinetics during pregnancy. Few studies have evaluated antituberculosis drug concentrations during pregnancy, which is commonly associated with modest reductions in the concentrations of other drugs. Conversely, rifampin clearance is reduced during pregnancy, possibly due to estrogen-related cholestasis [70]. Pharmacokinetics in infants are less predictable than in older children or adults due to growth and development. There is an important gap in evidence to support dosing in infants and pharmacokinetic studies are urgently needed [55].

## 5. Pharmacogenetic variation

Little is known about the role of pharmacogenetic variants in determining the disposition of antituberculosis drugs with the exception of isoniazid. Most studies investigating associations between antituberculosis drug concentrations and genetic variants have utilized a targeted approach evaluating specific single nucleotide polymorphism(SNP)s in relatively small studies. Barring *NAT2* for isoniazid, there is currently insufficient evidence on which to recommend dose optimization based on genotype.

The impact of mutations in *N-acetyl transferase 2(NAT2)* genes controlling conversion of isoniazid to acetyl-isoniazid in the intestinal and hepatic cells, is well known. A trimodal distribution of isoniazid exposures is described, although in many studies the respective distributions of the apparent clearance for intermediate and rapid metabolizers are not clearly distinguished [71–73]. The AUC among slow acetylators is typically 2- to 3-fold greater than in rapid acetylators.

Standard (4–6 mg/kg daily, in adults [40]) doses of isoniazid are effective and well tolerated in the majority of patients with drug susceptible tuberculosis. Although dosing stratified by *NAT2* genotype has resulted in lower rates of toxicity and improved efficacy in patient with DS-TB [74], it is in patients with DR-TB that stratified dosing would play a more important role. High doses of isoniazid (10–15 mg/kg daily) are necessary to overcome strains of *M. tuberculosis* strains with low levels of resistance to isoniazid, which are associated with *inhA* gene mutations [75–77]. However, slow acetylators are at risk of toxicity when increased doses of isoniazid are prescribed and rapid acetylators may need even higher doses for optimal efficacy [75–78]. Dose adjusting strategies have been proposed based on the presence of *inhA* or *katG* mutations in *Mycobacterium tuberculosis* [79]. Currently *NAT2* genotyping is not routinely available. The efficacy and toxicity of the proposed doses should be confirmed in slow and rapid acetylators, and the marked geographic variation in the distribution of *NAT2* genotypes should be considered when optimizing doses [76,77,79]. While several SNPs in the *NAT2* gene are used to categorize the genotype into slow, intermediate, and rapid acetylator types according to their number of functional *NAT2* alleles, the identification and validation of a *NAT2* tag SNP would simplify the development of genetic tests separating slow from intermediate and rapid acetylators [80,81]. Thus, if feasible and affordable genotyping methods were developed, a priori optimization of isoniazid doses by genotype could be contemplated.

The extent to which genetic variation governs the large differences in rifampin exposures observed between patients and between studies, is not well understood [30]. Organic-anion-transporting polypeptide1B1 (encoded by *SLCO1B1*) mediates transfer of rifampin into the hepatocyte, from where it is excreted via the bile. Studies describe associations between *SLCO1B1* variants and rifampin as well as rifabutin. However, as the findings have been inconsistent across the studies in different populations, further studies are needed to confirm the associations and identify particular single nucleotide polymorphisms related to rifampin pharmacokinetics [82–87]. Functional polymorphisms of the gene encoding arylacetamide deacetylase, which efficiently converts rifamycins to 25-desacetylrifamycin metabolites, have been identified, however their clinical relevance has not been confirmed [86,88]. Although rifampin is also a substrate of p-glycoprotein and it causes autoinduction regulated by the pregnane X receptor, pharmacokinetic associations with variants in the genes encoding these proteins have not been described [86]. Similarly, isolated findings which should be confirmed ascribe changes in moxifloxacin pharmacokinetics to SNPs in *SLCO1B1, UGT1A,* and *ABCB1* [89–91].

## 6. Pharmacokinetic drug-drug interactions

Drug-drug interactions are an important source of pharmacokinetic variability. The drug-drug interactions between tuberculosis treatment and the drugs used to treat HIV-infection and other common comorbidities such as diabetes and hypertension need to be characterized in the relevant patient populations and managed appropriately. That rifampin is a potent inducer of multiple drug metabolizing enzymes and transporters is well known and the effects of tuberculosis drugs on antiretrovirals administered concurrently has been highlighted [92,93]. The drug interactions affecting TB drug pharmacokinetics are less well recognized. Perpetrator drugs may be within the multidrug antituberculosis regimens or administered for comorbid conditions. The interactions may be unanticipated, particularly for some of the older drugs whose metabolic pathways have not been well characterized.

Ritonavir-boosted protease inhibitors potently inhibit multiple drug disposition pathways and induce others. Specifically, in settings with a high burden of tuberculosis, they are widely used as the key component of antiretroviral regimens in children under 3 years of age and in adults on second line treatment. Concomitantly administered ritonavir-boosted protease inhibitors result in markedly increased rifabutin exposures. As a result, the dose of rifabutin is reduced from 300 mg daily to 150 mg daily or three times a week in adults in order to mitigate exposure-related adverse effects [94–96]. The optimal dose in children has not been established [97]. While multiple studies have evaluated the effect of rifampin on antiretrovirals, few have evaluated rifampin concentrations. However, a study among TB/HIV co-infected patients in Mozambique found that neither efavirenz nor nevirapine affected rifampin concentrations [98]. Conversely, while the number of patients on second-line antiretroviral treatment regimens was small, a South African study suggests that rifampin exposures are increased by concomitant lopinavir/ritonavir in the doubled doses used to overcome the effect of rifampin on lopinavir concentrations [11].

Bedaquiline and its M2 metabolite are substrates of cytochrome p450 3A4. As bedaquiline has a very long terminal half-life, the extent of the efavirenz and lopinavir/ritonavir interactions with chronic administration were only appreciated when pharmacokinetic modeling was used to account the cumulative effects [99–101]. The 5-fold increase in bedaquiline’s clearance mediated by rifampin precludes their combined use [101]. Efavirenz also induces the metabolism of bedaquiline resulting in a 50% reduction in exposure with chronic use [99]. Consequently, combination with efavirenz is avoided. Conversely lopinavir/ritonavir inhibits bedaquiline’s metabolism resulting in a 3-fold increase in exposures with long-term administration, while M2 exposures are not substantially affected [100,102]. Models predict that this interaction may enhance efficacy without increasing toxicity, however, these findings should be confirmed in clinical studies [103,104]. As nevirapine has little impact on bedaquiline concentrations, it is preferred if the integrase inhibitor dolutegravir is not available.

In cases of low levels of resistance to isoniazid, some patients will fail to achieve sufficient isoniazid exposures especially if they are also rapid acetylators [77]. The 30% reduction in isoniazid exposures described by Bhatt et al. in HIV infected tuberculosis patients administered efavirenz-based antiretroviral treatment is therefore of concern [98]. The interaction has been confirmed in a second study among patients with TB/HIV coinfection, in which induction of the acetylation pathway was apparent, affecting predominantly rapid and intermediate acetylators [14]. Cycloserine has been reported to interfere with the absorption of isoniazid, but this effect is poorly understood [105]. A recent study described a 40% reduction in isoniazid in patients with multidrug-resistant tuberculosis(MDR-TB) when the drugs including terizidone (which consists of two linked molecules of cycloserine) are crushed and combined together with water [106]. That terizidone may reduce the bioavailability of isoniazid is supported by a recent study in children which described an 80% reduction in isoniazid exposures when it was administered as part of a terizidone-containing regimen for MDR-TB treatment and compared to the peak concentration and AUC in children on the same dose of isoniazid in a terizidone-sparing regimen for prevention of MDR-TB [107]. Studies to define within regimen drug-drug interactions are critical for selection of the optimal doses and drug combinations for emerging regimens to treat DR-TB.

Moxifloxacin is conjugated in the cytosol to glucuronide and sulfate metabolites. Concomitant rifampin reduces moxifloxacin AUC by about 30% [108], which is likely to be clinically important in patients on a daily dose of 400 mg daily [109]. This interaction may have contributed to the failure of regimens including moxifloxacin with rifampin, pyrazinamide, and isoniazid or ethambutol, to shorten the duration of tuberculosis treatment [110,111]. A recent study found that efavirenz-based ART, reduced moxifloxacin AUC by 30% in TB/HIV coinfected patients. During the cotreatment period, rifampin mediated an additional 30% reduction in moxifloxacin exposures [112]. The effect of efavirenz-mediated induction of UGT on moxifloxacin exposures may be critical for patients with drug-resistant tuberculosis. A recent study among South African patients with MDR-TB found that the conservative AUC/MIC target of 53 is not achieved in 15% and 75% of patients with moxifloxacin MICs 0.12 and 0.25 mg/L, respectively [49]. Should the substantial effect of efavirenz on moxifloxacin exposures be confirmed, adjusted doses of moxifloxacin or alternative treatment strategies should be evaluated.

While drug-drug interactions may be unanticipated, *in vitro* methods should be used to screen drug combinations for potential interactions. Combinations with potential for interaction should be confirmed and quantified in clinical pharmacokinetic studies. Ideally these should be conducted in patient populations, accounting for changes over time due to induction of pharmacokinetic pathways and accumulation with repeated doses, and in the context of the relevant environmental, dietary and genetic factors. Alternative dosing or drug strategies need to be sought for patients at risk of clinically significant drug-drug interactions. As the extent of drug-drug interactions in children may differ between children and adults, and can be modified by formulation effects, dose adjusting strategies employed to overcome an interaction should be studied using appropriate formulations in the relevant age groups [113].

## 7. Dose preparation and administration

When food considerably increases bioavailability, as for rifapentine, bedaquiline, delamanid, and clofazimine among other drugs, dosing advise is usually to take the medication with food [114–117]. However, the diet of patients varies considerably and as the burden of tuberculosis is greatest among the socio-economically disadvantaged, scant food may be available. It is therefore important that phase III studies (preferably with embedded pharmacokinetic-pharmacodynamic studies) evaluate the drug under realistic field conditions with respect to food intake.

Crushing of tablets before administration is common in young children as suitable formulations are frequently not available for those unable to swallow whole tablets. Adults on regimens involving the ingestion of multiple drugs may also prefer take their tablets crushed [106]. Crushing of tablets before mixing with water may reduce bioavailability as was demonstrated for rifapentine [118]. While formal studies of medicine administration practices in the home are scant, crushed tablets are frequently mixed together in water or another vehicle before swallowing. Drugs may then interact with other drugs or substances in the mixture, for example: some fluoroquinolones are chelated by divalent cations such as Fe++ and Ca++ [119,120]; certain sugars interact with the absorption of isoniazid [121]; and, as discussed above, terizidone may interfere with the absorption of isoniazid.

There is a critical need to develop affordable formulations suitable for children in high burden settings that also have reliable bioavailability under field conditions. Emerging techniques allow exploration of the potential for novel cost-efficient formulations to improve drug delivery. Nanoparticle formulations, for example, may potentially extend the product shelf-life, improve safety and tolerability, enhance the pharmacokinetic profile to optimize the key pharmacokinetic measures related to drug activity, and reduce pharmacokinetic variability [122]. Moreover, more efficient absorption into the systemic circulation may allow reduced content of the active ingredient, thereby making the drug more affordable. In the absence of formulations developed for solution, suspension or mixing with food, the available formulations should be investigated for their suitability to be manipulated for use in young children [123,124].

Appropriate FDC formulations are important for the long-term implementation of these multidrug regimens. The rational design of FDC combinations can be facilitated by model-based tools. Svensson et al. developed a methodology integrating information about the intended use population, the pharmacokinetic properties of the drugs, their therapeutic targets, and practical constraints which they applied to rifampin, isoniazid and pyrazinamide thus synthesizing an optimal FDC for children with tuberculosis [125]. Such tools can expedite more sustainable production of suitable formulations.

## 8. Laboratory error

While the extent to which inter-laboratory differences in the measurement of drug concentrations in plasma and serum contribute to pharmacokinetic variability between studies is poorly understood. Participation in proficiency testing schemes would facilitate attribution of pharmacokinetic variability between studies as well as pooling of studies to understand geographic, genetic and environmental differences between populations.

## 9. Conclusion

The pharmacokinetics of the antituberculosis drugs vary widely in patients on recommended doses. The optimal drug exposures based on pharmacokinetic-pharmacodynamic relationships within patients on multidrug regimens is the subject of ongoing research and is beyond the scope of this review. Here we have focussed on factors associated with variability in drug exposures between patients and study populations. In order to limit the number of patients at risk of a poor treatment response or toxicity due to low or high drug exposures, respectively, dosing should be adjusted by factors causing clinically important changes in drug exposures.

As smaller individuals have a higher dose requirement per kilogram of fat free mass, the currently recommended approach applying uniform mg/kg doses for the first-line regimen, results in lower drug exposures among small individuals, wasted patients, and males. We used model-based simulations to predict improved weight band-based doses of the first-line antituberculosis regimen using the currently available FDCs. While the potential effects of genetic variants on the disposition of most antituberculosis drugs has been incompletely evaluated, the effect of *NAT2* genotype on isoniazid concentrations is large and clinically important. Important drug-drug interactions affecting the concentrations of several antituberculosis drugs have been identified. If confirmed the effects of efavirenz on moxifloxacin and isoniazid exposures, and the effect terizidone/cycloserine on isoniazid exposures, are likely to be clinically important. Formulation quality, product preparation and dose administration practices are poorly appreciated sources of pharmacokinetic variability that can have a large effect on drug exposures. For children in particular, improved formulations should be developed. More effective assurance of formulation quality is needed.

## 10. Expert commentary

A growing body of literature provides evidence that the variability in drug concentrations among patients on standard regimens has clinically important consequences. We identified various factors which should be addressed to reduce the number of patients at risk of poor treatment response or toxicity due to low or high drug exposures respectively.

We propose a simplified approach to optimize dosing for patients with DS-TB. By using the widely available FDCs in the currently recommended weight bands the improved doses could be implemented efficiently. One additional FDC per dose in patients weighing under 55 kg results in more equitable drug exposures across the weight bands and to avoids underdosing of patients in the lowest two weight bands. Similarly, weight-based doses for children should be adjusted to avoid underdosing of children in the lower weight bands.

Several further aspects require further research as well as the development of approaches to address them:

Stratified dosing of isoniazid by *NAT2* genotype would improve dosing, especially in patients needing high doses of isoniazid to overcome low levels of resistance, however, testing of acetylator type is not available as part of routine care.

Drug-drug interactions can result in substantially altered drug exposure. They need to be identified and if clinically significant, adjusted dosing strategies should be studied in adults and children, respectively, unless suitable alternative regimens are available. Should the effect of efavirenz on moxifloxacin and isoniazid exposures and the effect terizidone/cycloserine on isoniazid exposures be confirmed, adjusted doses to overcome the interactions should be explored. The appropriate doses of rifabutin for TB/HIV infected children cotreated with a boosted protease inhibitor need to be defined.

Current mechanisms to assure the quality of antituberculosis drug formulations are not adequate to prevent the appearance of substandard products on the market and in treatment programs to an alarming extent. Feasible ways need to be developed and funded, to protect patients from substandard products.

New technologies provide opportunities to optimize formulations for adults and children. For many of the antituberculosis drugs, there is an urgent need for suitable pediatric formulations to be developed.

## 11. Five-year view

Global recognition of the burden of tuberculosis has led to a resurgence of research in the last two decades, which will improve the use of drugs in regimens used for treatment and to prevent transmission. Given its toll on children and pregnant women, optimization of drug use during pregnancy, breastfeeding, and the early years of life are imperative. The elderly are an expanding group neglected by pharmacokinetic research. The use of concomitant medicines to control noncommunicable diseases is likely to increase in high burden settings, hence the characterization and management of drug-drug interactions with frequently prescribed drugs for these conditions will become more urgent. The ongoing development of new drugs for tuberculosis and HIV will necessitate resources and capacity to ensure that drug-drug interactions are identified and appropriately addressed.

Methods used to study the clinical importance of pharmacokinetic variability in the context of multi-drug regimens are likely to mature and gain wider credibility. In addition to the application of modeling and simulations to optimize doses and drug combinations for best treatment response in patient cohorts, integration of *in vitro*, animal and clinical data is likely to be used more widely to support pharmacokinetic-pharmacodynamic models and extend their predictions to severe forms of disease (such as tuberculosis meningitis) and special populations. Studies evaluating the effects of drug pressure on changes in the mycobacterial genome and gene expression will improve our understanding of the importance pharmacokinetic variability in the emergence of drug resistance.

Thus evidence-based definition of target drug exposure ranges will support the development of dosing strategies achieving target concentrations in a higher proportion of patients. Moreover, for the less effective and more toxic second-line regimens, characterization of important pharmacokinetic-pharmacodynamic relationships for key drugs driving efficacy and/or toxicity may support the need for therapeutic drug concentration monitoring where this is available and the development of field-friendly tests to support wider use of individualized dosing.

Using currently available technologies, the development of better formulations for adults and children may enormously improve drug delivery through more efficient absorption and improved acceptability. Evidence of the cost effectiveness of improved formulations would support the necessary investments. None-the-less there will be an ongoing need for more effective measures to ensure the quality of antituberculosis drug products.

## Key issues

- The variability in drug concentrations among patients on standard regimens has clinically important consequences.
- Weight band-based doses for adults and children need to be adjusted to account for the increased clearance per kilogram of body weight in smaller people.
- Stratified dosing of isoniazid by *NAT2* genotype would improve dosing, especially in patients needing high doses of isoniazid to overcome *Mycobacterium tuberculosis* with low levels of isoniazid resistance.
- Drug-drug interactions can result in substantially altered drug exposure. They need to be identified, and if clinically significant adjusted dosing strategies should be studied.
- Renewed efforts are needed to assure the quality of antituberculosis drug formulations.
- New technologies should be leveraged to improve formulations for adults and children.

## References

[1] van Crevel R, Alisjahbana B, de Lange WC, et al Low plasma concentrations of rifampicin in tuberculosis patients in Indonesia. Int J Tuberc Lung Dis. 2002;6(6):497–502. 12068982 10.5588/09640569513002

[2] McIlleron H, Wash P, Burger A, et al Determinants of rifampin, isoniazid, pyrazinamide, and ethambutol pharmacokinetics in a cohort of tuberculosis patients. Antimicrob Agents Chemother. 2006;50(4):1170–1177. 16569826 10.1128/AAC.50.4.1170-1177.2006 PMC1426981

[3] Ruslami R, Nijland HM, Alisjahbana B, et al Pharmacokinetics and tolerability of a higher rifampin dose versus the standard dose in pulmonary tuberculosis patients. Antimicrob Agents Chemother. 2007;51(7):2546–2551. 17452486 10.1128/AAC.01550-06 PMC1913243

[4] Chideya S, Winston CA, Peloquin CA, et al Isoniazid, rifampin, ethambutol, and pyrazinamide pharmacokinetics and treatment outcomes among a predominantly HIV-infected cohort of adults with tuberculosis from Botswana. Clin Infect Dis. 2009;48(12):1685–1694. 19432554 10.1086/599040 PMC3762461

[5] McIlleron H, Rustomjee R, Vahedi M, et al Reduced antituberculosis drug concentrations in HIV-infected patients who are men or have low weight: implications for international dosing guidelines. Antimicrob Agents Chemother. 2012;56(6):3232–3238. 22411614 10.1128/AAC.05526-11 PMC3370773

[6] Pasipanodya JG, McIlleron H, Burger A, et al Serum drug concentrations predictive of pulmonary tuberculosis outcomes. J Infect Dis. 2013;208(9):1464–1473. 23901086 10.1093/infdis/jit352 PMC3789573

[7] Jeremiah K, Denti P, Chigutsa E, et al Nutritional supplementation increases rifampin exposure among tuberculosis patients coinfected with HIV. Antimicrob Agents Chemother. 2014;58(6):3468–3474. 24709267 10.1128/AAC.02307-13 PMC4068463

[8] Chigutsa E, Pasipanodya JG, Visser ME, et al Impact of nonlinear interactions of pharmacokinetics and MICs on sputum bacillary kill rates as a marker of sterilizing effect in tuberculosis. Antimicrob Agents Chemother. 2015;59(1):38–45. 25313213 10.1128/AAC.03931-14 PMC4291375

[9] Denti P, Jeremiah K, Chigutsa E, et al Pharmacokinetics of isoniazid, pyrazinamide, and ethambutol in newly diagnosed pulmonary tb patients in tanzania. PLoS One. 2015;10(10):e0141002. 26501782 10.1371/journal.pone.0141002 PMC4621059

[10] Chirehwa MT, Rustomjee R, Mthiyane T, et al Model-based evaluation of higher doses of rifampin using a semimechanistic model incorporating autoinduction and saturation of hepatic extraction. Antimicrob Agents Chemother. 2015;60(1):487–494. 26552972 10.1128/AAC.01830-15 PMC4704145

[11] Rockwood N, Meintjes G, Chirehwa M, et al HIV-1 Coinfection does not reduce exposure to rifampin, isoniazid, and pyrazinamide in south african tuberculosis outpatients. Antimicrob Agents Chemother. 2016;60(10):6050–6059. 27480859 10.1128/AAC.00480-16 PMC5038257

[12] Chirehwa MT, McIlleron HM, Wiesner L, et al Population pharmacokinetics of 1st-line antituberculosis drugs administered under three treatment strategies in TB/HIV patients from West Africa [abstract P_23]. 9th International Workshop on Clinical Pharmacology of Tuberculosis Drugs; 2016 10 24; Liverpool (United Kingdom).

[13] Chirehwa MT, McIlleron H, Rustomjee R, et al Pharmacokinetics of pyrazinamide and optimal dosing regimens for drug-sensitive and -resistant tuberculosis. Antimicrob Agents Chemother. 2017;61(8):e00490–17. 28607022 10.1128/AAC.00490-17 PMC5527644

[14] Chirehwa M, McIlleron H, Wiesner L, et al Effect of efavirenz-based antiretroviral therapy and high-dose rifampicin on the pharmacokinetics of isoniazid and acetyl-isoniazid. J Antimicrob Chemother. 2018 [Epub ahead of print] DOI:10.1093/jac/dky378. PMC6293084 30239829

[15] Prahl JB, Johansen IS, Cohen AS, et al Clinical significance of 2 h plasma concentrations of first-line anti-tuberculosis drugs: a prospective observational study. J Antimicrob Chemother. 2014;69(10):2841–2847. 25140577 10.1093/jac/dku210

[16] Mah A, Kharrat H, Ahmed R, et al Serum drug concentrations of INH and RMP predict 2-month sputum culture results in tuberculosis patients. Int J Tuberc Lung Dis. 2015;19(2):210–215. 25574921 10.5588/ijtld.14.0405

[17] Rockwood N, Pasipanodya JG, Denti P, et al Concentration-dependent antagonism and culture conversion in pulmonary tuberculosis. Clin Infect Dis. 2017;64(10):1350–1359. 28205671 10.1093/cid/cix158 PMC5411399

[18] Guiastrennec B, Ramachandran G, Karlsson MO, et al Suboptimal antituberculosis drug concentrations and outcomes in small and HIV-coinfected children in India: recommendations for dose modifications. Clin Pharmacol Ther. 2017 DOI:10.1002/cpt.987. PMC6004234 29247506

[19] Jindani A, Borgulya G, de Patiño IW, et al A randomised Phase II trial to evaluate the toxicity of high-dose rifampicin to treat pulmonary tuberculosis. Int J Tuberc Lung Dis. 2016;20(6):832–838. 27155189 10.5588/ijtld.15.0577

[20] Boeree MJ, Heinrich N, Aarnoutse R, et al High-dose rifampicin, moxifloxacin, and SQ109 for treating tuberculosis: a multi-arm, multi-stage randomised controlled trial. Lancet Infect Dis. 2017;17(1):39–49. 28100438 10.1016/S1473-3099(16)30274-2 PMC5159618

[21] Savic RM, Weiner M, MacKenzie WR, et al Defining the optimal dose of rifapentine for pulmonary tuberculosis: exposure-response relations from two phase II clinical trials. Clin Pharmacol Ther. 2017;102(2):321–331. 28124478 10.1002/cpt.634 PMC5545752

[22] Svensson RJ, Svensson EM, Aarnoutse RE, et al Greater early bactericidal activity at higher rifampicin doses revealed by modeling and clinical trial simulations. J Infect Dis. 2018;218(6):991–999. 29718390 10.1093/infdis/jiy242

[23] Velásquez GE, Brooks MB, Coit JM, et al Efficacy and safety of high-dose rifampin in pulmonary tuberculosis. a randomized controlled trial. Am J Respir Crit Care Med. 2018;198(5):657–666. 29954183 10.1164/rccm.201712-2524OC PMC6118011

[24] Njie GJ, Morris SB, Woodruff RY, et al Isoniazid-rifapentine for latent tuberculosis infection: a systematic review and meta-analysis. Am J Prev Med. 2018;55(2):244–252. 29910114 10.1016/j.amepre.2018.04.030 PMC6097523

[25] Achmad TH, van der Ven AJ, Borm G, et al Intensified regimen containing rifampicin and moxifloxacin for tuberculous meningitis: an open-label, randomised controlled phase 2 trial. Lancet Infect Dis. 2013;13(1):27–35. 23103177 10.1016/S1473-3099(12)70264-5

[26] Shenje J, Ifeoma Adimora-Nweke F, Ross IL, et al Poor penetration of antibiotics into pericardium in pericardial tuberculosis. EBioMedicine. 2015;2(11):1640–1649. 26870790 10.1016/j.ebiom.2015.09.025 PMC4740291

[27] Savic RM, Ruslami R, Hibma JE, et al Pediatric tuberculous meningitis: model-based approach to determining optimal doses of the anti-tuberculosis drugs rifampin and levofloxacin for children. Clin Pharmacol Ther. 2015;98(6):622–629. 26260983 10.1002/cpt.202 PMC4888594

[28] Bolhuis MS, Akkerman OW, Sturkenboom MG, et al Individualized treatment of multidrug-resistant tuberculosis using therapeutic drug monitoring. Int J Mycobacteriol. 2016;5(Suppl 1):S44–S45. 28043603 10.1016/j.ijmyco.2016.07.003

[29] Lange C, Alghamdi WA, Al-Shaer MH, et al Perspectives for personalized therapy for patients with multidrug-resistant tuberculosis. J Intern Med. 2018 5 28 [Epub ahead of print] DOI:10.1111/joim.12780. 29806961

[30] Stott KE, Pertinez H, Sturkenboom MGG, et al Pharmacokinetics of rifampicin in adult TB patients and healthy volunteers: a systematic review and meta-analysis. J Antimicrob Chemother. 2018;73(9):2305–2313. 29701775 10.1093/jac/dky152 PMC6105874

[31] Pillai G, Fourie PB, Padayatchi N, et al Recent bioequivalence studies on fixed-dose combination anti-tuberculosis drug formulations available on the global market. Int J Tuberc Lung Dis. 1999;3(11):S309–16. 10593710

[32] Hao LH, Guo SC, Liu CC, et al Comparative bioavailability of rifampicin and isoniazid in fixed-dose combinations and single-drug formulations. Int J Tuberc Lung Dis. 2014;18(12):1505–1512. 25517820 10.5588/ijtld.13.0647

[33] Sekaggya-Wiltshire C, Chirehwa M, Musaazi J, et al Low anti-tuberculosis drug concentrations in HIV-Tuberculosis co-infected adults with low body weight. [Abstract 39]. 11th International Workshop on Clinical Pharmacology of Tuberculosis Drugs; 2018 10 23; The Hague (Netherlands).

[34] McIlleron H, Wash P, Burger A, et al Widespread distribution of a single drug rifampicin formulation of inferior bioavailability in South Africa. Int J Tuberc Lung Dis. 2002;6(4):356–361. 11936746

[35] Milán-Segovia RC, Domínguez-Ramírez AM, Jung-Cook H, et al Relative bioavailability of rifampicin in a three-drug fixed-dose combination formulation. Int J Tuberc Lung Dis. 2010;14(11):1454–1460. 20937187

[36] McIlleron H, Hundt H, Smythe W, et al Bioavailability of two licensed paediatric rifampicin suspensions: implications for quality control programmes. Int J Tuberc Lung Dis. 2016;20(7):915–919. 27287644 10.5588/ijtld.15.0833 PMC4978631

[37] Court R, Chirehwa MT, Wiesner L, et al Quality assurance of rifampicin-containing fixed-drug combinations in South Africa: dosing implications. Int J Tuberc Lung Dis. 2018 5 1;22(5):537–543. 29663959 10.5588/ijtld.17.0697 PMC5905389

[38] Survey of the quality of antituberculosis medicines circulating in selected newly independent states of the former Soviet Union. Geneva: World Health Organization; 2011 (WHO/EMP/QSM/2011.2) Accessed 2018 828 Available from: https://extranet.who.int/prequal/sites/default/files/documents/TBQuality-Survey_Nov2011_1.pdf

[39] Singh S, Mohan B. A pilot stability study on four-drug fixed-dose combination anti-tuberculosis products. Int J Tuberc Lung Dis. 2003;7(3):298–303. 12661847

[40] Treatment of tuberculosis: guidelines. 4th ed. Geneva: World Health Organization; 2010 (WHO/HTM/TB/2009.420) Accessed 2018 914 Available from: http://www.who.int/tb/publications/2010/9789241547833/en/23741786

[41] Guidance for national tuberculosis programmes on the management of tuberculosis in children. 2nd ed. Geneva: World Health Organization; 2014 (WHO/HTM/TB/2014.03) Accessed 2018 829 Available from: http://www.who.int/tb/publications/childtb_guidelines/en/24999516

[42] Denti P, Gonzalez-Martinez C, Winckler J, et al Pharmacokinetics of rifampicin in African children: evaluation of the new WHO dosing guidelines [abstract OA-155–13]. 48th World Conference on Lung Health of the International Union Against Tuberculosis and Lung Disease; 2017 10 11–14; Guadalajara (Mexico). Int J Tuberc Lung Dis. 2017;21(11):S203.

[43] Horita Y, Alsultan A, Kwara A, et al evaluation of the adequacy of who revised dosages of the first-line antituberculosis drugs in children with tuberculosis using population pharmacokinetic modeling and simulations. Antimicrob Agents Chemother. 2018;62(9):e00008–18. 29914960 10.1128/AAC.00008-18 PMC6125554

[44] McIlleron H, Chabala C, Wiesner L, et al Rifampicin and pyrazinamide exposures in children with DS-TB on WHO-recommended FDCs in the SHINE trial [abstract oa22–340–27]. 49th UnionWorld Conference on Lung Health; 2018 10 24–27; The Hague (Netherlands). Int J Tuberc Lung Dis. 2018;22(11):S441.

[45] Holford N, Heo YA, Anderson B A pharmacokinetic standard for babies and adults. J Pharm Sci. 2013;102(9):2941–2952. 23650116 10.1002/jps.23574

[46] Mfinanga SG, Kirenga BJ, Chanda DM, et al Early versus delayed initiation of highly active antiretroviral therapy for HIV-positive adults with newly diagnosed pulmonary tuberculosis (TB-HAART): a prospective, international, randomised, placebo-controlled trial. Lancet Infect Dis. 2014;14(7):563–571. 24810491 10.1016/S1473-3099(14)70733-9

[47] Griesel R, Stewart A, van der Plas H, et al Prognostic indicators in the World Health Organization’s algorithm for seriously ill HIV-infected inpatients with suspected tuberculosis. AIDS Res Ther. 2018;15(1):5. 29433509 10.1186/s12981-018-0192-0 PMC5808414

[48] Javaid A, Ahmad N, Afridi AK, et al validity of time to sputum culture conversion to predict cure in patients with multidrug-resistant tuberculosis: a retrospective single-center study. Am J Trop Med Hyg. 2018;98(6):1629–1636. 29611497 10.4269/ajtmh.17-0936 PMC6086179

[49] Chirehwa MT, Court R, De Kock M, et al Moxifloxacin population pharmacokinetics and exposure/MIC target attainment in patients on treatment for MDR-TB [Abstract 21]. 11th International Workshop on Clinical Pharmacology of Tuberculosis Drugs; 2018 10 23; The Hague (Netherlands).

[50] Wasserman S, Denti P, Brust J, et al Clofazimine pharmacokinetics in South African patients with drug-resistant tuberculosis [Abstract 9]. 11th International Workshop on Clinical Pharmacology of Tuberculosis Drugs; 2018 10 23; The Hague (Netherlands).

[51] Alsultan A, Peloquin CA Therapeutic drug monitoring in the treatment of tuberculosis: an update. Drugs. 2014;74(8):839–854. 24846578 10.1007/s40265-014-0222-8

[52] Kearns GL, Abdel-Rahman SM, Alander SW, et al Developmental pharmacology–drug disposition, action, and therapy in infants and children. N Engl J Med. 2003;349(12):1157–1167. 13679531 10.1056/NEJMra035092

[53] Zvada SP, Denti P, Donald PR, et al Population pharmacokinetics of rifampicin, pyrazinamide and isoniazid in children with tuberculosis: in silico evaluation of currently recommended doses. J Antimicrob Chemother. 2014;69(5):1339–1349. 24486870 10.1093/jac/dkt524 PMC3977610

[54] Hiruy H, Rogers Z, Mbowane C, et al Subtherapeutic concentrations of first-line anti-TB drugs in South African children treated according to current guidelines: the PHATISA study. J Antimicrob Chemother. 2015;70(4):1115–1123. 25505005 10.1093/jac/dku478 PMC4356201

[55] Bekker A, Schaaf HS, Draper HR, et al Pharmacokinetics of rifampin, isoniazid, pyrazinamide, and ethambutol in infants dosed according to revised who-recommended treatment guidelines. Antimicrob Agents Chemother. 2016;60(4):2171–2179. 26810651 10.1128/AAC.02600-15 PMC4808214

[56] Rapid advice: treatment of tuberculosis in children. Geneva: World Health Organization; 2010 (WHO/HTM/TB/2010.13) Accessed 2018 830 Available from: http://apps.who.int/iris/bitstream/handle/10665/44444/9789241500449_eng.pdf?sequence=1 26269860

[57] McIlleron H, Chirehwa M, Chabala C, et al Rifampicin, isoniazid and pyrazinamide exposures in children with DS-TB on WHO-recommended FDCs in the SHINE trial [Abstract 6]. 11th International Workshop on Clinical Pharmacology of Tuberculosis Drugs; 2018 23 10; The Hague (Netherlands).

[58] Thee S, Seifart HI, Rosenkranz B, et al Pharmacokinetics of ethionamide in children. Antimicrob Agents Chemother. 2011;55(10):4594–4600. 21788468 10.1128/AAC.00379-11 PMC3186973

[59] Thee S, Garcia-Prats AJ, Draper HR, et al Pharmacokinetics and safety of moxifloxacin in children with multidrug-resistant tuberculosis. Clin Infect Dis. 2015;60(4):549–556. 25362206 10.1093/cid/ciu868 PMC4366580

[60] Garcia-Prats AJ, Draper HR, Thee S, et al Pharmacokinetics and safety of ofloxacin in children with drug-resistant tuberculosis. Antimicrob Agents Chemother. 2015;59(10):6073–6079. 26195507 10.1128/AAC.01404-15 PMC4576031

[61] Denti P, Garcia-Prats AJ, Draper HR, et al Levofloxacin population pharmacokinetics in South African children treated for multidrug-resistant tuberculosis. Antimicrob Agents Chemother. 2018;62(2):e01521–17. 29133560 10.1128/AAC.01521-17 PMC5786780

[62] Gurumurthy P, Ramachandran G, Hemanth Kumar AK, et al Malabsorption of rifampin and isoniazid in HIV-infected patients with and without tuberculosis. Clin Infect Dis. 2004;38:280–283. 14699462 10.1086/380795

[63] Gurumurthy P, Ramachandran G, Hemanth Kumar AK, et al Decreased bioavailability of rifampin and other antituberculosis drugs in patients with advanced human immunodeficiency virus disease. Antimicrob Agents Chemother. 2004;48:4473–4475. 15504887 10.1128/AAC.48.11.4473-4475.2004 PMC525439

[64] Pinheiro VG, Ramos LM, Monteiro HS, et al Intestinal permeability and malabsorption of rifampin and isoniazid in active pulmonary tuberculosis. Braz J Infect Dis. 2006;10:374–379. 17420908 10.1590/s1413-86702006000600003

[65] Facanha MC, Gondim AM, Pinheiro VG, et al Intestinal barrier function and serum concentrations of rifampin, isoniazid and pyrazinamide in patients with pulmonary tuberculosis. Braz J Infect Dis. 2009;13:210–217. 20191199 10.1590/s1413-86702009000300011

[66] McIlleron H, Watkins ML, Folb PI, et al Rifampin levels, interferon-gamma release and outcome in complicated pulmonary tuberculosis. Tuberculosis (Edinb). 2007;87(6):557–564. 17890156 10.1016/j.tube.2007.08.002

[67] Vinnard C, Manley I, Scott B, et al A pilot study of immune activation and rifampin absorption in hiv-infected patients without tuberculosis infection: a short report. Tuberc Res Treat. 2017;2017:2140974. 29430306 10.1155/2017/2140974 PMC5752984

[68] Vinnard C, Ravimohan S, Tamuhla N, et al Markers of gut dysfunction do not explain low rifampicin bioavailability in HIV-associated TB. J Antimicrob Chemother. 2017;72(7):2020–2027. 28472448 10.1093/jac/dkx111 PMC5890696

[69] Alghamdi WA, Al-Shaer MH, Peloquin CA Protein binding of first-line antituberculosis drugs. Antimicrob Agents Chemother. 2018;62(7):e00641–18. 29735566 10.1128/AAC.00641-18 PMC6021678

[70] Denti P, Martinson N, Cohn S, et al Population pharmacokinetics of rifampin in pregnant women with tuberculosis and HIV coinfection in soweto, South Africa. Antimicrob Agents Chemother. 2015;60(3):1234–1241. 26643345 10.1128/AAC.02051-15 PMC4776013

[71] Parkin DP, Vandenplas S, Botha FJ, et al Trimodality of isoniazid elimination: phenotype and genotype in patients with tuberculosis. Am J Respir Crit Care Med. 1997;155(5):1717–1722. 9154882 10.1164/ajrccm.155.5.9154882

[72] Verhagen LM, Coenen MJ, López D, et al Full-gene sequencing analysis of NAT2 and its relationship with isoniazid pharmacokinetics in Venezuelan children with tuberculosis. Pharmacogenomics. 2014;15(3):285–296. 24533708 10.2217/pgs.13.230

[73] Hemanth Kumar AK, Ramesh K, Kannan T, et al N-acetyltransferase gene polymorphisms & plasma isoniazid concentrations in patients with tuberculosis. Indian J Med Res. 2017;145(1):118–123. 28574024 10.4103/ijmr.IJMR_2013_15 PMC5460557

[74] Azuma J, Ohno M, Kubota R, et al NAT2 genotype guided regimen reduces isoniazid-induced liver injury and early treatment failure in the 6-month four-drug standard treatment of tuberculosis: a randomized controlled trial for pharmacogenetics-based therapy. Eur J Clin Pharmacol. 2013;69(5):1091–1101. 23150149 10.1007/s00228-012-1429-9 PMC3641305

[75] Katiyar SK, Bihari S, Prakash S, et al A randomised controlled trial of high-dose isoniazid adjuvant therapy for multidrug-resistant tuberculosis. Int J Tuberc Lung Dis. 2008 2;12(2):139–145. 18230245

[76] Cordes H, Thiel C, Aschmann HE, et al A physiologically based pharmacokinetic model of isoniazid and its application in individualizing tuberculosis chemotherapy. Antimicrob Agents Chemother. 2016;60(10):6134–6145. 27480867 10.1128/AAC.00508-16 PMC5038291

[77] Zuur MA, Pasipanodya JG, van Soolingen D, et al Intermediate susceptibility dose-dependent breakpoints for high dose rifampicin, isoniazid and pyrazinamide treatment in multidrug-resistant tuberculosis programmes. Clin Infect Dis. 2018 4 24 DOI:10.1093/cid/ciy346. 29697766

[78] Shi J, Xie M, Wang J, et al Susceptibility of N-acetyltransferase 2 slow acetylators to antituberculosis drug-induced liver injury: a meta-analysis. Pharmacogenomics. 2015;16(18):2083–2097. 26616266 10.2217/pgs.15.144

[79] Technical report on the pharmacokinetics and pharmacodynamics (PK/PD) of medicines used in the treatment of drug-resistant tuberculosis. Geneva: World Health Organization; 2018 (WHO/CDS/TB/2018.6) Accessed 2018 92 Available from: http://apps.who.int/iris/bitstream/handle/10665/260440/WHO-CDS-TB-2018.6-eng.pdf?sequence=1&isAllowed=y

[80] Ho HT, Wang TH, Hsiong CH, et al The NAT2 tag SNP rs1495741 correlates with the susceptibility of antituberculosis drug-induced hepatotoxicity. Pharmacogenet Genomics. 2013;23(4):200–207. 23407048 10.1097/FPC.0b013e32835e95e1

[81] McCallum AD, Schipani A, Owen A, et al Genetic determinants of the pharmacokinetic variability of isoniazid and rifampicin in Malawian adults with pulmonary tuberculosis [abstract OA-422–05]. 46th World Conference on Lung Health of the International Union Against Tuberculosis and Lung Disease; 2015 12 2–6; Cape Town (South Africa). Int J Tuberc Lung Dis. 2015;19(12):S293.

[82] Weiner M, Peloquin C, Burman W, et al Effects of tuberculosis, race, and human gene SLCO1B1 polymorphisms on rifampin concentrations. Antimicrob Agents Chemother. 2010;54(10):4192–4200. 20660695 10.1128/AAC.00353-10 PMC2944564

[83] Chigutsa E, Visser ME, Swart EC, et al The SLCO1B1 rs4149032 polymorphism is highly prevalent in South Africans and is associated with reduced rifampin concentrations: dosing implications. Antimicrob Agents Chemother. 2011;55(9):4122–4127. 21709081 10.1128/AAC.01833-10 PMC3165308

[84] Gengiah TN, Botha JH, Soowamber D, et al Low rifampicin concentrations in tuberculosis patients with HIV infection. J Infect Dev Ctries. 2014;8(8):987–993. 25116663 10.3855/jidc.4696

[85] Hennig S, Naiker S, Reddy T, et al Effect of SLCO1B1 polymorphisms on rifabutin pharmacokinetics in African HIV-infected patients with tuberculosis. Antimicrob Agents Chemother. 2015;60(1):617–620. 26482301 10.1128/AAC.01195-15 PMC4704238

[86] Sloan DJ, McCallum AD, Schipani A, et al Genetic ##. Antimicrob Agents Chemother. 2017 6 27;61(7):e00210–17. 28461315 10.1128/AAC.00210-17 PMC5487625

[87] Dompreh A, Tang X, Zhou J, et al Effect of genetic variation of nat2 on isoniazid and SLCO1B1 and CES2 on rifampin pharmacokinetics in ghanaian children with tuberculosis. Antimicrob Agents Chemother. 2018 2 23;62(3):e02099–17. 29263072 10.1128/AAC.02099-17 PMC5826147

[88] Shimizu M, Fukami T, Kobayashi Y, et al A novel polymorphic allele of human arylacetamide deacetylase leads to decreased enzyme activity. Drug Metab Dispos. 2012;40:1183–1190. 22415931 10.1124/dmd.112.044883

[89] Weiner M, Burman W, Luo CC, et al Effects of rifampin and multidrug resistance gene polymorphism on concentrations of moxifloxacin. Antimicrob Agents Chemother. 2007;51(8):2861–2866. 17517835 10.1128/AAC.01621-06 PMC1932492

[90] Naidoo A, Ramsuran V, Chirehwa M, et al Effect of genetic variation in UGT1A and ABCB1 on moxifloxacin pharmacokinetics in South African patients with tuberculosis. Pharmacogenomics. 2018;19(1):17–29. 29210323 10.2217/pgs-2017-0144 PMC5753622

[91] Weiner M, Gelfond J, Johnson-Pais TL, et al Elevated plasma moxifloxacin concentrations and SLCO1B1 g.-11187G>A polymorphism in adults with pulmonary tuberculosis. Antimicrob Agents Chemother. 2018;62(5):e01802–17. 29463526 10.1128/AAC.01802-17 PMC5923103

[92] McIlleron H, Meintjes G, Burman WJ, et al Complications of antiretroviral therapy in patients with tuberculosis: drug interactions, toxicity, and immune reconstitution inflammatory syndrome. J Infect Dis. 2007;196(Suppl 1):S63–75. 17624828 10.1086/518655

[93] Maartens G, Boffito M, Flexner CW Compatibility of next-generation first-line antiretrovirals with rifampicin-based antituberculosis therapy in resource limited settings. Curr Opin HIV AIDS. 2017;12(4):355–358. 28403028 10.1097/COH.0000000000000376

[94] Naiker S, Connolly C, Wiesner L, et al Randomized pharmacokinetic evaluation of different rifabutin doses in African HIV- infected tuberculosis patients on lopinavir/ritonavir-based antiretroviral therapy. BMC Pharmacol Toxicol. 2014;15:61. 25406657 10.1186/2050-6511-15-61 PMC4277828

[95] Hennig S, Svensson EM, Niebecker R, et al Population pharmacokinetic drug-drug interaction pooled analysis of existing data for rifabutin and HIV PIs. J Antimicrob Chemother. 2016;71(5):1330–1340. 26832753 10.1093/jac/dkv470 PMC4830412

[96] Lan NT, Thu NT, Barrail-Tran A, et al Randomised pharmacokinetic trial of rifabutin with lopinavir/ritonavir-antiretroviral therapy in patients with HIV-associated tuberculosis in Vietnam. PLoS One. 2014;9(1):e84866. 24465443 10.1371/journal.pone.0084866 PMC3898920

[97] Moultrie H, McIlleron H, Sawry S, et al Pharmacokinetics and safety of rifabutin in young HIV-infected children receiving rifabutin and lopinavir/ritonavir. J Antimicrob Chemother. 2015;70(2):543–549. 25281400 10.1093/jac/dku382 PMC4291235

[98] Bhatt NB, Barau C, Amin A, et al Pharmacokinetics of rifampin and isoniazid in tuberculosis-HIV-coinfected patients receiving nevirapine- or efavirenz-based antiretroviral treatment. Antimicrob Agents Chemother. 2014;58(6):3182–3190. 24663014 10.1128/AAC.02379-13 PMC4068429

[99] Svensson EM, Aweeka F, Park JG, et al Model-based estimates of the effects of efavirenz on bedaquiline pharmacokinetics and suggested dose adjustments for patients coinfected with HIV and tuberculosis. Antimicrob Agents Chemother. 2013;57(6):2780–2787. 23571542 10.1128/AAC.00191-13 PMC3716161

[100] Svensson EM, Dooley KE, Karlsson MO Impact of lopinavir-ritonavir or nevirapine on bedaquiline exposures and potential implications for patients with tuberculosis-HIV coinfection. Antimicrob Agents Chemother. 2014;58(11):6406–6412. 25114140 10.1128/AAC.03246-14 PMC4249405

[101] Svensson EM, Murray S, Karlsson MO, et al Rifampicin and rifapentine significantly reduce concentrations of bedaquiline, a new anti-TB drug. J Antimicrob Chemother. 2015;70(4):1106–1114. 25535219 10.1093/jac/dku504 PMC4356204

[102] Brill MJ, Svensson EM, Pandie M, et al Confirming model-predicted pharmacokinetic interactions between bedaquiline and lopinavir/ritonavir or nevirapine in patients with HIV and drug-resistant tuberculosis. Int J Antimicrob Agents. 2017;49(2):212–217. 28038962 10.1016/j.ijantimicag.2016.10.020

[103] Svensson EM, Karlsson MO Modelling of mycobacterial load reveals bedaquiline’s exposure-response relationship in patients with drug-resistant TB. J Antimicrob Chemother. 2017;72(12):3398–3405. 28961790 10.1093/jac/dkx317 PMC5890768

[104] Tanneau L, Svensson EM, Rossenu S, et al Bedaquiline appears to antagonize its own main metabolite’s QTcF interval prolonging effect [abstract 8634; www.page-meeting.org/?abstract=8634]. PAGE 27; 2018 May 29-June 1; Montreux (Switzerland): PAGE. Abstracts of the Annual Meeting of the Population Approach Group in Europe. ISSN 1871–6032.

[105] TB Alliance Cycloserine. Tuberculosis. 2008;88:100–101. 18486041 10.1016/S1472-9792(08)70007-6

[106] Court R, Chirehwa MT, Wiesner L, et al Bioavailability of pyrazinamide, moxifloxacin, isoniazid, ethambutol, and terizidone when tablets are crushed in the treatment of multidrug-resistant tuberculosis [Abstract 25]. 11th International Workshop on Clinical Pharmacology of Tuberculosis Drugs; 2018 10 23; The Hague (Netherlands).

[107] Winckler JL, Schaaf SH, Draper HR, et al The pharmacokinetics of high dose Isoniazid for the prevention or treatment of drug-resistant tuberculosis in HIV-infected and -uninfected children [abstract ep04–128–26]. 49th UnionWorld Conference on Lung Health; 2018 10 24–27; The Hague (Netherlands). Int J Tuberc Lung Dis. 2018;22(11):S314.

[108] Nijland HM, Ruslami R, Suroto AJ, et al Rifampicin reduces plasma concentrations of moxifloxacin in patients with tuberculosis. Clin Infect Dis. 2007;45(8):1001–1007. 17879915 10.1086/521894

[109] Gumbo T, Louie A, Deziel MR, et al Selection of a moxifloxacin dose that suppresses drug resistance in Mycobacterium tuberculosis, by use of an in vitro pharmacodynamic infection model and mathematical modeling. J Infect Dis. 2004;190(9):1642–1651. 15478070 10.1086/424849

[110] Gillespie SH, Crook AM, McHugh TD, et al Four-month moxifloxacin-based regimens for drug-sensitive tuberculosis. N Engl J Med. 2014;371(17):1577–1587. 25196020 10.1056/NEJMoa1407426 PMC4277680

[111] Jawahar MS, Banurekha VV, Paramasivan CN, et al Randomized clinical trial of thrice-weekly 4-month moxifloxacin or gatifloxacin containing regimens in the treatment of new sputum positive pulmonary tuberculosis patients. PLoS One. 2013;8(7):e67030. 23843980 10.1371/journal.pone.0067030 PMC3700922

[112] Naidoo A, Chirehwa M, McIlleron H, et al Effect of rifampicin and efavirenz on moxifloxacin concentrations when co-administered in patients with drug-susceptible TB. J Antimicrob Chemother. 2017;72(5):1441–1449. 28175315 10.1093/jac/dkx004 PMC5890691

[113] Zhang C, Denti P, Decloedt EH, et al Model-based evaluation of the pharmacokinetic differences between adults and children for lopinavir and ritonavir in combination with rifampicin. Br J Clin Pharmacol. 2013;76(5):741–751. 23432610 10.1111/bcp.12101 PMC3853533

[114] Nix DE, Adam RD, Auclair B, et al Pharmacokinetics and relative bioavailability of clofazimine in relation to food, orange juice and antacid. Tuberculosis (Edinb). 2004;84(6):365–373. 15525560 10.1016/j.tube.2004.04.001

[115] Zvada SP, Van Der Walt JS, Smith PJ, et al Effects of four different meal types on the population pharmacokinetics of single-dose rifapentine in healthy male volunteers. Antimicrob Agents Chemother. 2010;54(8):3390–3394. 20516273 10.1128/AAC.00345-10 PMC2916304

[116] European Medicines Agency Sirturo product information. Product information. EMEA/H/C/002614 -R/0024; [updated 05/03/2018; cited 2018 8 29]. Available from: http://www.ema.europa.eu/docs/en_GB/document_library/EPAR_-_Product_Information/human/002614/WC500163209.pdf

[117] European Medicines Agency Deltyba product information. Product information. EMEA/H/C/002552 -IAIN/0031. 11/06/2018; [cited 2018 8 29]. Available from: http://www.ema.europa.eu/docs/en_GB/document_library/EPAR_-_Product_Information/human/002552/WC500166232.pdf

[118] Weiner M, Savic RM, Kenzie WR, et al Rifapentine pharmacokinetics and tolerability in children and adults treated once weekly with rifapentine and isoniazid for latent tuberculosis infection. J Pediatric Infect Dis Soc. 2014;3(2):132–145. 26625366 10.1093/jpids/pit077

[119] Neuvonen PJ, Kivistö KT, Lehto P Interference of dairy products with the absorption of ciprofloxacin. Clin Pharmacol Ther. 1991;50(5 Pt 1):498–502. 1934862 10.1038/clpt.1991.174

[120] Stass H, Kubitza D Effects of iron supplements on the oral bioavailability of moxifloxacin, a novel 8-methoxyfluoroquinolone, in humans. Clin Pharmacokinet. 2001;40(Suppl 1):57–62. 11352443 10.2165/00003088-200140001-00008

[121] Rao KV, Kailasam S, Menon NK, et al Inactivation of isoniazid by condensation in a syrup preparation. Bull World Health Organ. 1971;45(5):625–632. 5316955 PMC2427966

[122] Giardiello M, Liptrott NJ, McDonald TO, et al Accelerated oral nanomedicine discovery from miniaturized screening to clinical production exemplified by paediatric HIV nanotherapies. Nat Commun. 2016;7:13184. 27767027 10.1038/ncomms13184 PMC5078733

[123] Svensson EM, Du Bois J, Kitshoff R, et al Relative bioavailability of bedaquiline tablets suspended in water: implications for dosing in children. Br J Clin Pharmacol. 2018 DOI:10.1111/bcp.13696. PMC6138504 29952141

[124] Harausz EP, Leigh J, Garcia-Prats AJ, et al Stability of second-line tuberculosis medications mixed with milk or yogurt. Clin Infect Dis. 2017;65(4):704–705. 10.1093/cid/cix434 28482036

[125] Svensson EM, Yngman G, Denti P, et al Evidence-based design of fixed-dose combinations: principles and application to pediatric anti-tuberculosis therapy. Clin Pharmacokinet. 2018;57(5):591–599. 28779464 10.1007/s40262-017-0577-6 PMC5904239

