# Supplementary material for: Current research toward optimizing dosing of first-line antituberculosis treatment
Source: Expert Rev Anti Infect Ther. 2018 Dec 12;17(1):27–38. doi: 10.1080/14787210.2019.1555031 (PMC6364307; doi:10.1080/14787210.2019.1555031)
Supplement: Supplemental Material [file IERZ_A_1555031_SM5103.docx]

**Appendix: Description of the population pharmacokinetic models used for the simulations**

Population pharmacokinetic models were developed using data collected from the TB‑HAART and RAFA studies, and a description of these studies has been reported [1][2]. Demographic data of a reference cohort of TB patients (n = 1092), with or without HIV coinfection recruited in clinical trials conducted in West Africa and South Africa were used for the simulations. The pharmacokinetic models for rifampin and pyrazinamide were developed using the concentration‑time data collected from the TBHAART study. Blood samples for quantification of drug concentrations were collected on four PK sampling days (day 1, 8, 15 and 29) from 61 patients. Rifampin pharmacokinetics was best described a semi-mechanistic model incorporating autoinduction and saturation of hepatic extraction [3]. The prediction-corrected visual predictive check (Figure 2) shows that the model adequately described the pharmacokinetics of rifampin and simulations of exposures corresponding to higher doses of rifampin up to 35 mg/kg closely mirrored those obtained in clinical trials. The steady‑state parameter estimates from the final model were used to simulate rifampin exposures presented in this report (Table 1). A one-compartment disposition model with first-order absorption and elimination best described the pharmacokinetics of pyrazinamide. Clearance of pyrazinamide increased linearly from the first day of treatment to day 29 (14% increase). Parameter estimates of the final model on day 29 (~steady‑state) of treatment were used to simulate pyrazinamide exposures presented in this report (Table 1). Allometric scaling was applied to all clearance and volume of distribution parameters using fat-free mass (standardized to the fat-free mass of 42 kg, the median value of the cohort) which was superior to total body weight.

Parameter estimates of the population pharmacokinetic models for isoniazid and ethambutol developed using concentration-time data from the RAFA study were used to simulates the isoniazid and ethambutol exposures. Blood sampling for pharmacokinetic analysis was done at between four to eight weeks after the start of tuberculosis treatment. Samples were collected before the dose and at 2, 4, 6 and 10 hours post‑dose on the sampling day. The model describing the pharmacokinetics of isoniazid (n = 150) and acetyl-isoniazid (n = 79) has been reported [2]. The pharmacokinetics of isoniazid was described by a two-compartment disposition model with first-order absorption and elimination and a liver compartment to capture the first-pass effect. Two elimination pathways were defined: one for the formation of acetyl-isoniazid and another for the formation of other metabolites and/or excretion. The parameter estimates of clearance presented in Table 1 are an aggregate of the two pathways stratified by acetylator status. A mixture model was implemented in the model to estimate separate acetylation clearance parameters for fast and slow acetylators of isoniazid. Each patient was randomly assigned to an acetylator group in each simulation replicate. The effect of body size was included in the model via allometric scaling using fat-free mass which resulted in a better fit compared with total body weight regardless of acetylators status. The model also detected a 54% increase in clearance of isoniazid among fast acetylators only on the elimination pathway associated with the formation of acetyl-isoniazid. The parameter estimates of the final model are presented in Table 1, and the model diagnostics are presented in Figure 2 of a previous report [2].

Concentration-time data for ethambutol were available in 222 patients from the RAFA study. The data were interpreted using nonlinear mixed-effects modelling in NONMEM software version 7.4 by using the algorithm first-order conditional estimation with eta-epsilon interaction. The models included both fixed-effect and random-effect parameters. A log-normal distribution was assumed for the random-effect parameters. The residual unexplained error model comprised both additive and proportional components. The pharmacokinetics of ethambutol was described using a two-compartment disposition model with first-order absorption and elimination. A delay in absorption of ethambutol was described using a chain of transit compartments. Ethambutol clearance was separated between renal and nonrenal components and the later was modulated by creatinine clearance (CrCl), computed using the Cockcroft-gault formula [4]. Allometric scaling was applied to all clearance and volume parameters using fat-free mass. Allometric scaling using total body weight did not result in a worse fit, but fat-free mass was chosen to maintain consistency with pharmacokinetic models of other drugs in the FDC. Relative bioavailability was fixed to a reference value of 1, and the data supported both between-subject and between-occasion variability. An individual with a bioavailability of 1.2 means that the bioavailability of the patient is 20% more compared with a typical individual. Parameter estimates of the final model used for the simulations are presented in Table 1. The volume and clearance parameters refer to a typical individual in the cohort with a fat-free mass of 43.3 kg. Figure 3 shows that the model adequately described the data.

Table 1 Estimated parameter values from the final models

| **Parameter** | **Estimate** | | | |
| --- | --- | --- | --- | --- |
|  | **Rifampin**^a^ | **Isoniazid**^b^ | **Pyrazinamide**^a^ | **Ethambutol**^b^ |
| Clearance: day 1 (L/h)^c^ | 93.2^d^ | - | 3.35 | - |
| Clearance: steady-state (L/h)^c^ | 176^d^ | 18.0^e^ | 3.83 | 40.6 |
| Clearance, renal: steady-state (L/h/75 mL/min CrCl) | - | - | - | 12.9 |
| Central volume (L)^c^ | 50.1 | 47.7 | 43.2 | 225 |
| Absorption rate constant (/h) | 1.96 | 1.59 | 3.54 | 1.56 |
| Mean transit time / Lag time (h) | 0.71 | 0.287 | 0.542 | 1.25 |
| Number of transit compartments | 19.3 | - | 28 | 15.7 |
| Bioavailability | 1 fixed | 1 fixed | 1 fixed | 1 fixed |
| Induction half-life (days) | 4.5 | - | - | - |
| Fraction unbound^f^ | 0.2 fixed | 0.95 fixed | - | - |
| Michaelis constant (mg/L) | 3.35 | - | - | - |
| Peripheral volume (L)^c^ | - | 8.14 | - | 415 |
| Intercompartmental clearance (L/h)^c^ | - |  | - | 47.2 |
|  |  |  |  |  |
| **Covariates**^g^ |  |  |  |  |
| Change in clearance for fast acetylators status (%) | - | +263 | - | - |
| Efavirenz effect on acetylation clearance in fast acetylators (%) | - | +54.1 | - | - |
|  |  |  |  |  |
| **Between subject variability** (%) |  |  |  |  |
| *Clearance* | 22.5 | 35.6 | 16.3 | 15.4 |
| *Central volume* | 14.2 | 18.9 | - | - |
| *Bioavailability* | - | - | 10.7 | 21.7 |
| *Absorption rate* | - | 36.7 | - | 84.7 |
| *Mean transit time* |  |  |  | 24.5 |
| **Between occasion variability (**%) |  |  |  |  |
| *Clearance* | 21.9 | - | 13.3 | - |
| *Bioavailability* | 11.0 | 16.9 | 11.9 | 17.5 |
| *Absorption rate* | 81.2 | - | 84.0 | - |
| *Mean transit time* | 62.7 | - | 52.9 | - |
| **Error** |  |  |  |  |
| Additive (mg/L) | 0.064 | 0.02 fixed | 1.23 | 0.0224 |
| Coefficient of variation (%) | 10.8 | 11.2 | 4.4 | 13.8 |

^a^TBHAART study.

^b^RAFA study.

^c^This parameter has been adjusted by allometric scaling, and the values reported refer

to a subject with an FFM of 42 kg (TBHAART study) or 43.3 kg (RAFA study).

^d^Maximum intrinsic clearance.

^e^Intrinsic clearance.

^f^Volume of liver and hepatic plasma flow were fixed to 1L and 50 L/h respectively, and allometrically scaled.

^g^Selected covariate used for simulating exposure presented. Other covariates reported in previous publications were set to zero including the effect of efavirenz effect on acetylation clearance in fast acetylators.

| 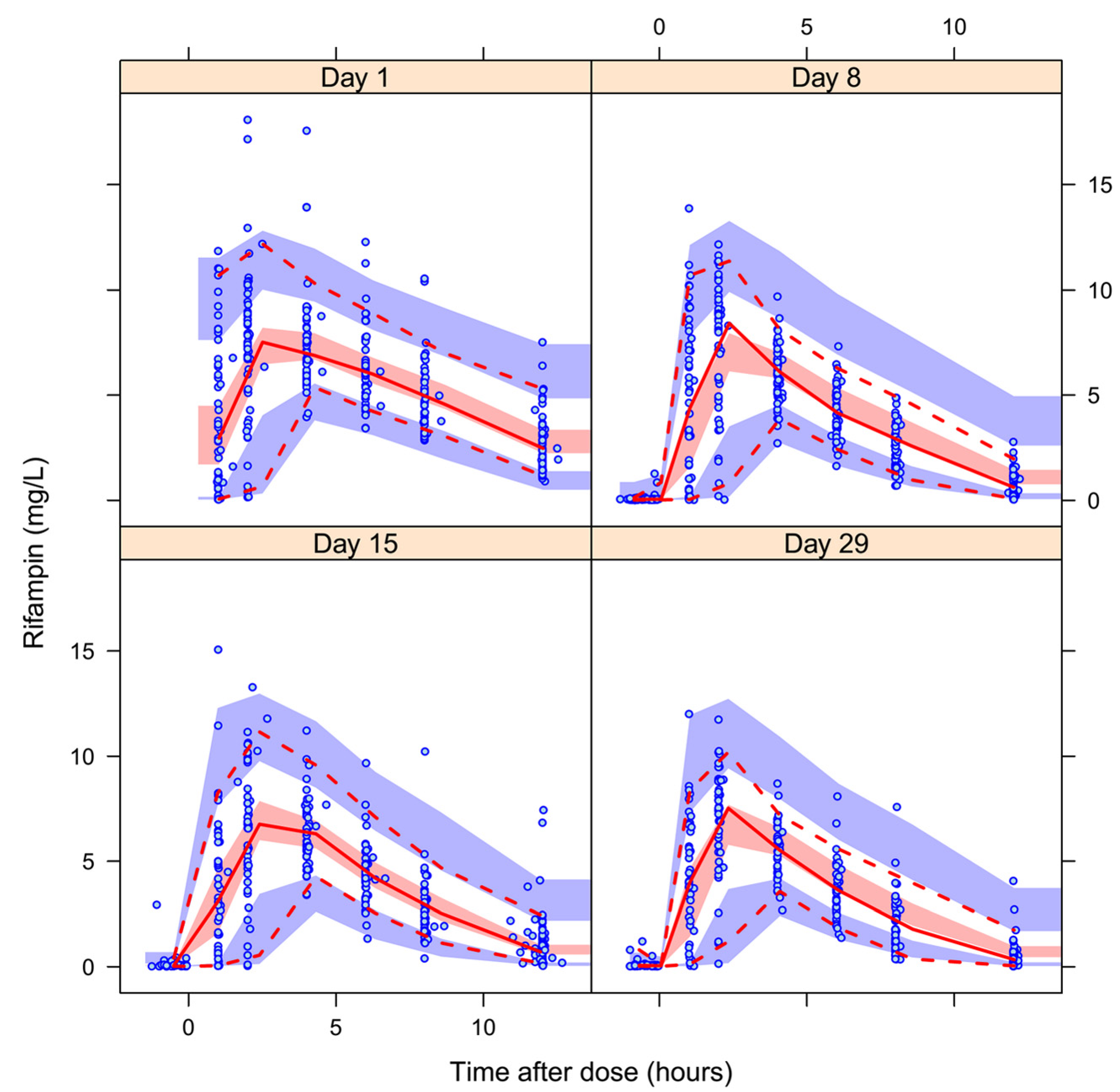 |
| --- |
| Figure 2 Prediction-corrected visual predictive check for a model describing the pharmacokinetics of rifampin stratified by days after the start of TB treatment. |

| 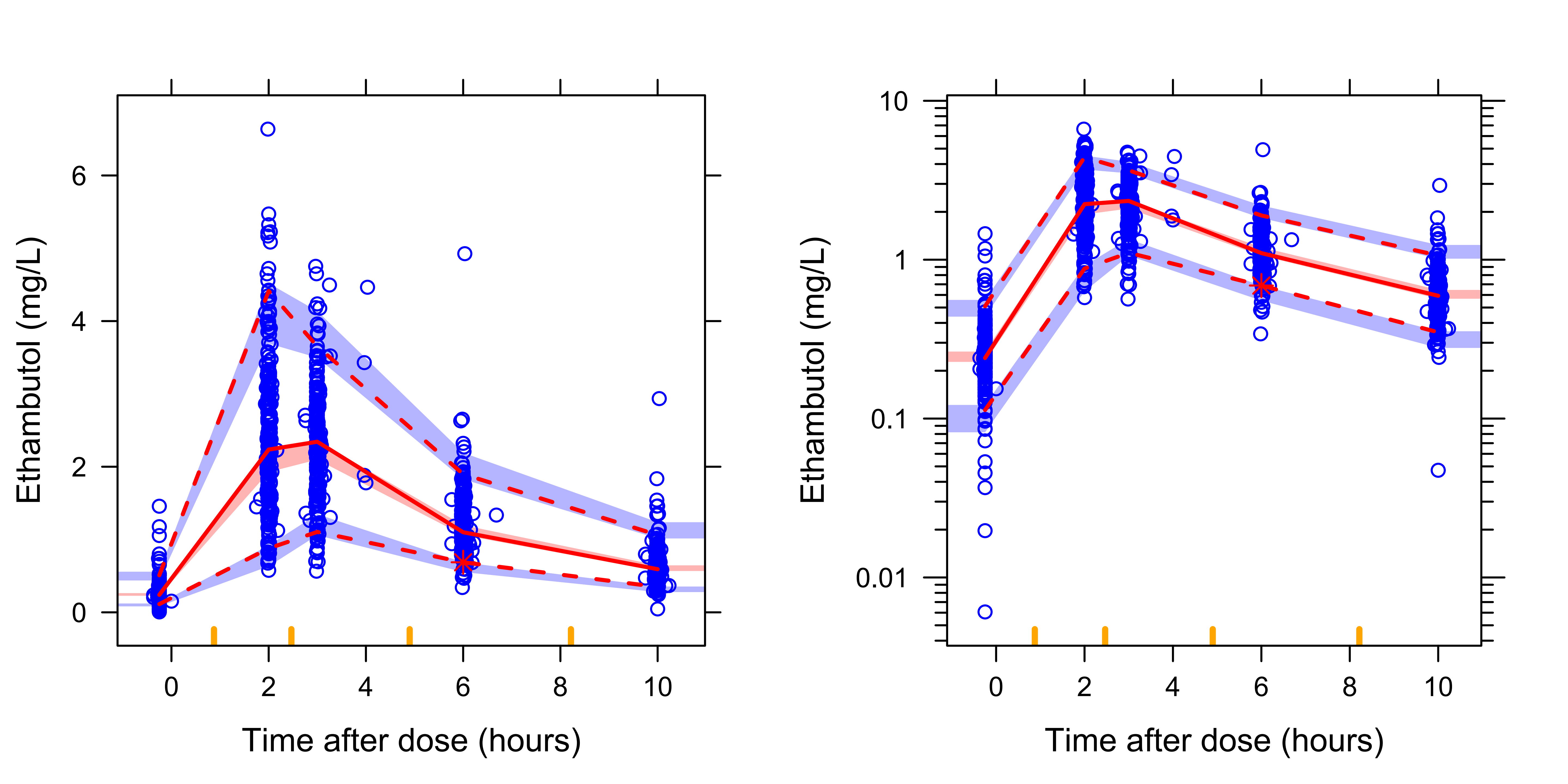 |
| --- |
| Figure 3 Prediction-corrected visual predictive check for a model describing the pharmacokinetics of ethambutol.  The left panel is the VPC in the normal scale and right panel in the VPC in the semi-log scale.  Simulations were used to predict the number of 4-drug FDC tablets (using the widely available rifampin/isoniazid/pyrazinamide/ethambutol 150/75/400/275 mg FDC) to achieve the most equitable drug exposures across weight bands. The distributions of weight, height and sex among 1092 African patients with drug sensitive tuberculosis in our database, on which the simulations were based, are described in Table 2.  Table 2: Weight, height, fat-free mass, and sex on which the simulations were based (N = 1092).   \| **Weight band** \| **30-37.4 kg** \| **37.5-54.4 kg** \| **54.5-70.4 kg** \| **>70.5 kg** \| \| --- \| --- \| --- \| --- \| --- \| \| No. of patients, n(%) \| 38 (3.5) \| 582 (53.5) \| 429 (39.2) \| 43 (3.8) \| \| Weight, kg^a^ \| 35.7 (30 – 37.4) \| 48.2 (37.5 – 54.4) \| 59.1 (54.5 – 70.4) \| 76.1 (70.5 - 102) \| \| Height, m^a^ \| 1.54 (1.44 – 1.80) \| 1.63 (1.35 – 1.93) \| 1.70 (1.41 – 1.98) \| 1.73 (1.42 – 1.94) \| \| Fat-free mass, kg^a^ \| 29.5 (24.2 – 34.3) \| 37.4 (26.4 – 49.1) \| 47.4 (35.1 – 60.2) \| 50.1 (38.9 – 71.7) \| \| Females, n (%) \| 17 (44.7) \| 268 (45.9) \| 171 (40.1) \| 24 (55.8) \|   ^a^ median (minimum, maximum) |

[1] McIlleron H, Rustomjee R, Vahedi M, et al. Reduced antituberculosis drug concentrations in HIV-infected patients who are men or have low weight: implications for international dosing guidelines. Antimicrob. Agents Chemother. 2012;56:3232–3238.

[2] Chirehwa MT, McIlleron H, Wiesner L, et al. Effect of efavirenz-based antiretroviral therapy and high-dose rifampicin on the pharmacokinetics of isoniazid and acetyl-isoniazid. J. Antimicrob. Chemother. 2018;In press.

[3] Chirehwa MT, Rustomjee R, Mthiyane T, et al. Model-Based Evaluation of Higher Doses of Rifampin Using a Semimechanistic Model Incorporating Autoinduction and Saturation of Hepatic Extraction. Antimicrob. Agents Chemother. 2016;60:487–494.

[4] Cockcroft DW, Gault H. Prediction of Creatinine Clearance from Serum Creatinine. Nephron. 1976;16:31–41.
